# Longitudinal Changes of NF-κB Downstream Mediators and Peritoneal Transport Characteristics in Incident Peritoneal Dialysis Patients

**DOI:** 10.1038/s41598-020-63258-3

**Published:** 2020-04-15

**Authors:** Winston Wing-Shing Fung, Peter Yam-Kau Poon, Jack Kit-Chung Ng, Vickie Wai-Ki Kwong, Wing-Fai Pang, Bonnie Ching-Ha Kwan, Phyllis Mei-Shan Cheng, Philip Kam-Tao Li, Cheuk-Chun Szeto

**Affiliations:** 1 Carol and Richard Yu Peritoneal Dialysis Research Centre, Department of Medicine & Therapeutics, Prince of Wales Hospital, The Chinese University of Hong Kong, Shatin, Hong Kong China; 2 Li Ka Shing Institute of Health Sciences (LiHS), Faculty of Medicine, The Chinese University of Hong Kong, Shatin, Hong Kong China

**Keywords:** Physiology, Nephrology

## Abstract

The role of intra-peritoneal mediators in the regulation peritoneal transport is not completely understood. We investigate the relation between longitudinal changes in dialysis effluent level of nuclear factor kappa-B (NF-κB) downstream mediators and the change in peritoneal transport over 1 year. We studied 46 incident PD patients. Their peritoneal transport characteristics were determined after starting PD and then one year later. Concomitant dialysis effluent levels of interleukin-6 (IL-6), cyclo-oxygenase-2 (COX-2) and hepatocyte growth factor (HGF) are determined. There were significant correlations between baseline and one-year dialysis effluent IL-6 and COX-2 levels with the corresponding dialysate-to-plasma creatinine level at 4 hours (D/P4) and mass transfer area coefficient of creatinine (MTAC). After one year, patients who had peritonitis had higher dialysis effluent IL-6 (26.6 ± 17.4 vs 15.1 ± 12.3 pg/ml, p = 0.037) and COX-2 levels (4.97 ± 6.25 vs 1.60 ± 1.53 ng/ml, p = 0.007) than those without peritonitis, and the number of peritonitis episode significantly correlated with the IL-6 and COX-2 levels after one year. In contrast, dialysis effluent HGF level did not correlate with peritoneal transport. There was no difference in any mediator level between patients receiving conventional and low glucose degradation product solutions. Dialysis effluent IL-6 and COX-2 levels correlate with the concomitant D/P4 and MTAC of creatinine. IL-6 and COX-2 may contribute to the short-term regulation of peritoneal transport.

## Introduction

Peritoneal dialysis (PD) is a life-saving treatment for patients with end stage kidney disease. There is a growing interest in promoting PD usage around the globe due to its technical simplicity and cost-effectiveness^1,2^.

The semi-permeable peritoneal membrane is the basis of successful PD. The peritoneal membrane consists of three major components, namely a monolayer of mesothelial cells, an interstitial layer of fibroblasts and a network of capillaries, but it is generally accepted that the endothelial membrane of the peritoneal capillaries is the main barrier to peritoneal transport^3,4^. With prolonged PD, exposure to bio-incompatible PD solutions, or infective insults, peritoneal membrane undergo inflammatory changes, neovascularization, and progressive fibrosis, eventually leads to peritoneal failure^5,6^.

The mechanisms of regulating peritoneal vascularity, inflammation and fibrosis are not completely understood, but nuclear factor kappa-B (NF-κB), the key pro-inflammatory transcriptional factor, appears to play a critical role in the regulation of inflammation, angiogenesis, and vascular permeability of the peritoneal membrane^7^. More recently, several downstream mediators are found to be responsible for the regulation of peritoneal transport by NF-κB. Notably, interleukin-6 (IL-6) is responsible for the control of small solute transport across the peritoneal membrane^8,9^. Cyclooxygenase-2 (COX-2) contributes to the short-term regulation of vascular smooth muscle tone, peritoneal transport of small solute as well as ultrafiltration^10^. On the other hand, hepatocyte growth factor (HGF) takes part in the long-term modulation of peritoneal fibrosis^11^.

However, the relation between these mediators and the corresponding alteration in peritoneal transport have not been fully elucidated. In the present study, we determine the relation between serial change in intra-peritoneal mediator levels and the concomitant alterations in peritoneal transport characteristics in incident PD patients.

## Patients and Methods

The study was approved by The Joint Chinese University of Hong Kong – New Territories East Cluster Clinical Research Ethics Committee (reference number CREC-2016.535). All studies procedures were in compliance with the Declaration of Helsinki. We studied 46 incident PD patients who participated in a prospective observational study that was previously reported^12^. All patients had the first peritoneal equilibration test (PET) and dialysis adequacy assessment around 4 weeks after PD training was completed. After one year of PD, PET was repeated. The clinical management of individual patient was decided by their nephrologists and not affected by the study. Clinical data were collected by medical record review. The Charlson’s Comorbidity Index (CCI) was used to assess the comorbidity load. Peritonitis episodes during the one year of observational period were recorded.

### Peritoneal equilibration test

The standard PET was used to assess the peritoneal transport characteristics^13^. All patients were in an euvolemic state with no peritonitis in the past one month. The procedure of standard PET has been described in detail previously^13^. The dialysate-to-plasma ratio of creatinine at 4 hours (D/P4), after correction of glucose interference^14^, was computed. Mass transfer area coefficients (MTAC) of creatinine normalized for body surface area (BSA) was calculated by a standard formula^15^:

BSA (m^2^) = 0.007184 × body weight (kg) ^0.425^ × body height (cm) ^0.725^

MTAC = V_e_ × [ln (V_0_ × Cr_b_) − ln (Cr_b_ − Cr_4_)] × 1000 ÷ 240 ÷ BSA × 1.73

where V_0_ and V_e_ were instilled volume (2 liters) and the volume of peritoneal dialysate effluent respectively. Cr_b_ and Cr_4_ were the creatinine concentration in plasma (at 2 hour) and in PDE at 4 hour respectively.

### Cytokine levels in PD effluent

Samples of PD effluent were collected at baseline and one year after PD during PET. We measured the PD effluent levels of interleukin-6 (IL-6), hepatocyte growth factor (HGF), and cyclooxygenase-2 (COX-2) by commercially available enzyme-linked immunosorbent assays (ELISA). These targets were selected because of their potential impact on peritoneal fibrosis and angiogenesis^9–11^. ELISA kits for IL-6 and HGF were purchased from R&D Systems (Minneapolis, MN), and kits for COX-2 from RayBiotech Inc. (Norcross, GA). All assay procedures were performed according to manufacturers’ instruction. The detection limits of IL-6, HGF, and COX-2 were 0.7 pg/ml, 40 pg/mL, and 1.2 ng/ml, respectively. All targets were assayed in duplicates.

### Clinical outcome

The primary outcome measures were the changes of peritoneal transport status over 1 year, as assessed by D/P4 and MTAC. The effect of peritonitis and type of PD solution on PD effluent cytokine levels are also explored. Peritonitis episode was defined according to the International Society for Peritoneal Dialysis (ISPD) guideline^16^. PD solutions are classified as conventional glucose based solutions and low glucose degradation product (GDP) ones.

### Statistical analysis

Statistical analysis was performed using the Statistical Package for Social Science version 24 (SPSS Inc., Chicago, IL). Correlation between PD effluent cytokine levels with peritoneal transport function and clinical parameters was calculated by the Spearman’s rank correlation coefficient. Longitudinal change in parameters after one year was compared by paired Student’s *t*-test or Wilcoxon signed rank test as appropriate. The PD effluent cytokines level and the peritoneal transport parameter among the different peritoneal dialysate systems was also compared by the independent Student’s t-test and Mann-Whitney U test as appropriate. A p-value of less than 0.05 was considered statistically significant. All probabilities were two-tailed.

## Human Rights

### Ethical approval:

All procedures performed in studies involving human participants were in accordance with the ethical standards of the institutional research committee at which the studies were conducted and with the 1964 Helsinki declaration and its later amendments or comparable ethical standards. The study was approved by the Clinical Research Ethics Committee of the Chinese University of Hong Kong (IRB approval number CRE-2016.535).

### Informed Consent

Written informed consent was obtained from all individual participants included in the study.

## Result

### Baseline demographic and clinical data

We recruited 46 incident PD patients (24 males). The average age was 58.2 ± 12.3 years; Charlson’s comorbidity index 5.5 ± 2.4. Their baseline and follow up clinical and biochemical data are summarized in Table 1. There was a strong internal correlation between baseline PD effluent IL-6 and COX-2 levels (r = 0.781, p < 0.01). In contrast, PD effluent HGF did not correlate with IL-6 or COX-2 levels.

**Table 1:** Baseline and follow up clinical and biochemical data.

	Baseline	1 year	P value
Body weight (kg)	65.4 ± 11.0	68.5 ± 11.8	p < 0.01*
Body mass index (kg/m^2^)	24.8 ± 3.3	25.9 ± 3.8	p < 0.01*
**Blood pressure (mmHg)**			
Systolic	146 ± 19	146 ± 20	p = 0.8*
Diastolic	75 ± 11	76 ± 13	p = 0.1*
Haemoglobin (g/dL)	9.8 ± 1.6	9.8 ± 1.8	p = 0.9*
Serum albumin (g/L)	34.9 ± 3.6	35.7 ± 2.9	p = 0.5*
**Peritoneal transport**			
D/P4	0.64 ± 0.13	0.66 ± 0.14	p = 0.3*
MTAC (ml/min/1.73m^2^)	9.37 ± 4.38	9.95 ± 5.02	p = 0.4*
UF volume (L)	0.33 ± 0.22	0.32 ± 0.19	p = 0.9*
Residual GFR (ml/min/1.73m^2^)	3.87 ± 2.23	2.84 ± 2.10	p = 0.01*
Weekly Kt/V	2.15 ± 0.60	1.96 ± 0.58	p = 0.1*
**PDE cytokines level**			
IL-6 (pg/ml)	23.28 ± 36.89	18.32 ± 14.66	p = 0.8**
COX-2 (pg/ml)	3.30 ± 5.21	2.58 ± 3.85	p = 0.1**
HGF (pg/ml)	228.0 ± 275.2	299.2 ± 590.8	p = 0.1**

D/P4, dialysate-to-plasma creatinine ratio at 4 hours; MTAC, mass transfer area coefficient of creatinine; UF, ultrafiltration; PDE, peritoneal dialysis effluent; IL-6, interleukin-6; COX-2, cyclooxygenases-2; HGF, hepatocyte growth factor. Data were compared by *paired Student’s t test or **Wilcoxon signed rank test.

### Changes over one year

Taken as a whole group, there was no significant change of D/P4, MTAC, or ultrafiltration volume over one year (Table 1). There was a modest but statistically significant correlation between baseline and one-year ultrafiltration volumes (r = 0.351, p = 0.02). In contrast, there was no significant correlation between baseline and one-year D/P4 (r = −0.018, p = 0.9) or MTAC (r = −0.044, p = 0.8).

There was no significant change of PD effluent IL-6, COX-2, or HGF level over one year (Table 1). Baseline and one-year PD effluent levels of IL-6 (r = 0.328, p = 0.03) and COX-2 (r = 0.358, p = 0.02) were significantly correlated, but the correlation between baseline and one-year PD effluent HGF level did not reach statistical significance (r = 0.340, p = 0.2). The change of PD effluent IL-6 level significantly correlate with the concomitant change of COX-2 level (r = 0.482, p < 0.01).

### Relation with peritoneal transport

**Figure 1:**
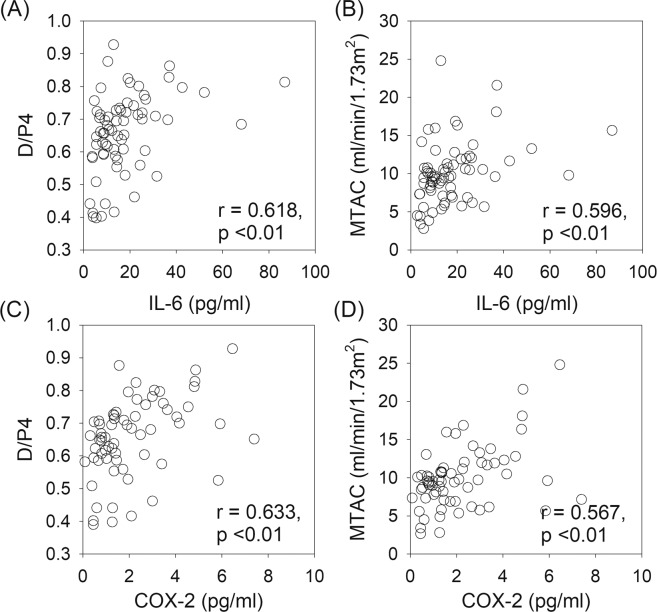
Correlations between baseline peritoneal dialysis (PD) effluent level of interleukin-6 (IL-6) and cyclooxygenase-2 (COX-2) with dialysate-to-plasma creatinine ratio at 4 hours (D/P4) and mass transfer area coefficient (MTAC) of creatinine. Data are compared by the Spearman’s rank correlation coefficient.

The relation between PD effluent cytokine levels and peritoneal transport parameters are summarized in Table 2. In essence, baseline PD effluent IL-6 and COX-2 levels had significant correlation with baseline D/P4 and MTAC creatinine (Fig. 1), but not ultrafiltration volume. Similarly, PD effluent IL-6 and COX-2 levels at one year had significant correlation with D/P4 and MTAC, but not ultrafiltration volume, at 1 year. In contrast, PD effluent HGF level did not correlate with any peritoneal transport parameter.

**Table 2:** Relation between PD effluent cytokines level and peritoneal transport characteristics.

	Baseline			1 year		
	IL-6	COX-2	HGF	IL-6	COX-2	HGF
baseline						
D/P4	r = 0.618, p < 0.01	r = 0.633, p < 0.01	r = −0.051, p = 0.7			
MTAC	r = 0.596, p < 0.01	r = 0.567, p < 0.01	r = −0.027, p = 0.9			
UF volume	r = 0.071, p = 0.7	r = 0.026, p = 0.9	r = −0.041, p = 0.8			
1 year						
D/P4	r = −0.067, p = 0.7	r = 0.013, p = 0.9	r = 0.035, p = 0.8	r = 0.648, p < 0.01	r = 0.721, p < 0.01	r = 0.201, p = 0.2
MTAC	r = −0.071, p = 0.7	r = −0.014, p = 0.9	r = 0.065, p = 0.7	r = 0.629, p < 0.01	r = 0.667, p < 0.01	r = 0.247, p = 0.1
UF volume	r = −0.059, p = 0.7	r = 0.091, p = 0.6	r = −0.362, p = 0.01	r = −0.056, p = 0.7	r = −0.044, p = 0.8	r = −0.040, p = 0.8

PD, peritoneal dialysis; D/P4, dialysate-to-plasma creatinine ratio at 4 hours; MTAC, mass transfer area coefficient of creatinine; UF, ultrafiltration, IL-6, interleukin-6; COX-2, cyclooxygenases-2; HGF, hepatocyte growth factor.

The change in PD effluent IL-6 and COX-2 levels from baseline to one year after PD also showed a significant correlation with the change in peritoneal transport, in terms of D/P4 and MTAC, during the same time (Table 3). On the other hand, change in PD effluent HGF level did not show any significant correlation with the change in D/P4 or MTAC. The change in ultrafiltration volume did not have significant correlation with the change in any PD effluent cytokine level. Baseline PD effluent IL-6 and COX-2 levels also correlate with the change in peritoneal transport over one year, but the correlation was less marked (Table 3).

**Table 3:** Relation between PD effluent cytokines level and change in peritoneal transport after 1 year.

Change in peritoneal transport	PD effluent cytokine level					
	Baseline level			Change over 1 year		
	IL-6	COX-2	HGF	IL-6	COX-2	HGF
D/P4	r = −0.491, p = 0.001	r = −0.466, p = 0.003	r = 0.052, p = 0.7	r = −0.709, p < 0.001	r = −0.555, p < 0.001	r = −0.168, p = 0.3
MTAC	r = −0.532, p < 0.001	r = −0.518, p = 0.001	r = 0.024, p = 0.9	r = −0.747, p < 0.001	r = −0.594, p < 0.001	r = −0.183, p = 0.2
UF volume	r = −0.122, p = 0.4	r = 0.028, p = 0.9	r = −0.382, p = 0.01	r = −0.059, p = 0.7	r = 0.030, p = 0.9	r = 0.039, p = 0.8

PD, peritoneal dialysis; D/P4, dialysate-to-plasma creatinine ratio at 4 hours; MTAC, mass transfer area coefficient of creatinine; UF, ultrafiltration, IL-6, interleukin-6; COX-2, cyclooxygenases-2; HGF, hepatocyte growth factor.

### Effect of peritonitis

**Figure 2:**
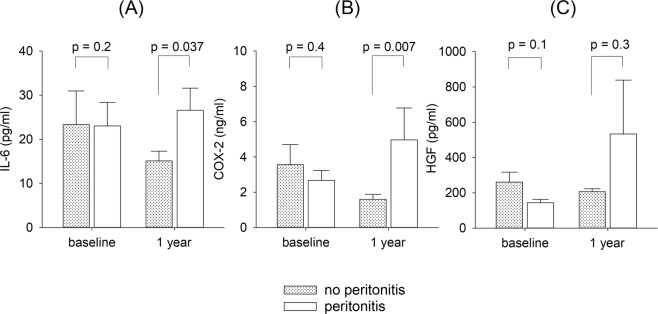
Comparison of peritoneal dialysis (PD) effluent level of (**A**) interleukin-6 (IL-6); (**B**) cyclooxygenase-2 (COX-2); and (**C**) hepatocyte growth factor (HGF) at baseline and one year after dialysis between patients with and without peritonitis.

During the one year of follow up period, 13 patients had 19 episodes of peritonitis; 33 patients remained peritonitis-free. There was no significant difference in baseline PD effluent IL-6 (23.0 ± 18.3 vs 23.4 ± 42.2 pg/ml, p = 0.9), COX-2 (143.5 ± 73.1 vs 261.3 ± 316.9 ng/ml, p = 0.2) or HGF levels (2.67 ± 1.94 vs 3.56 ± 6.09 pg/ml, p = 0.6) between patients with and without peritonitis episode in the subsequent one year. However, patients who had peritonitis during the follow up period had higher PD effluent IL-6 level (26.6 ± 17.4 vs 15.1 ± 12.3 pg/ml, p = 0.037) and COX-2 level (4.97 ± 6.25 vs 1.60 ± 1.53 ng/ml, p = 0.007) after one year than patients without peritonitis (Fig. 2). The number of peritonitis episode during follow up period also significantly correlated with the PD effluent IL-6 (r = 0.305, p = 0.047) and COX-2 levels (r = 0.374, p = 0.016) after one year. Similarly, patients who had peritonitis during the follow up period had higher D/P4 (0.741 ± 0.125 vs 0.628 ± 0.138, p = 0.026) and MTAC (12.96 ± 6.82 vs 8.77 ± 3.60 ml/min/1.73m^2^, p = 0.055) after one year than those without peritonitis, although the difference in MTAC did not reach statistical significance.

On the other hand, there was no significant difference in PD effluent HGF level one year after dialysis between patients with and without peritonitis episode (533.9 ± 1096.5 vs 206.7 ± 93.6 pg/ml, p = 0.3) (Fig. 2). However, patients with peritonitis had significantly more increase in PD effluent HGF level from baseline to one year than those without peritonitis (390.3 ± 1121.4 vs −54.6 ± 305.9 pg/ml, p = 0.024), and the number of peritonitis episode during follow up period had a modest but statistically significant correlation with the change in PD effluent HGF level over one year (r = −0.303, p = 0.041).

### Effect of dialysis solution

**Figure 3:**
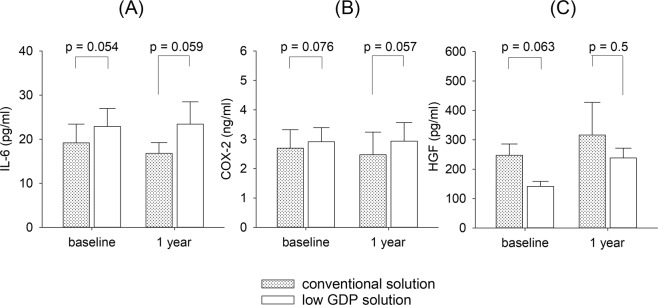
Comparison of peritoneal dialysis (PD) effluent level of (**A**) interleukin-6 (IL-6); (**B**) cyclooxygenase-2 (COX-2); and (**C**) hepatocyte growth factor (HGF)at baseline and one year after dialysis between patients treated with conventional PD solutions and low glucose degradation product (GDP) solutions.

Of the 46 patients, 44 had continuous ambulatory peritoneal dialysis with double bag system (34 had conventional PD solutions, 10 had low GDP solutions) and 2 had machine-assisted PD with conventional PD solutions. Patients who received low GDP solutions had marginally higher PD effluent IL-6 and COX-2, and lower HGF levels than patients who received conventional solutions, both at baseline and one year later (Fig. 3). However, none of the difference was statistically significant. In this study, there was no significant difference in D/P4, MTAC, or ultrafiltration volume between patients who received conventional and low GDP solutions, either at baseline or one year later (details not shown).

## Discussion

In the present study, we observed that the PD effluent IL-6 and COX-2 levels significantly associated with the concomitant D/P4 and MTAC of creatinine, and the change in these cytokines levels correlates with the corresponding alterations in these peritoneal transport parameters. Similar finding has been previously reported^8,9,17^. For example, dialysate IL-6 level was noted to be increased with longer PD duration, and dialysate IL-6 level was an independent predictor of peritoneal transport rate^9^. In the GLOBAL fluid study^17^, dialysate IL-6 level was the most significant predictor of the peritoneal transport rate. Clinical data on peritoneal COX-2 level are limited. However, COX-2 level has been shown to be increased in peritoneal mesothelial cells after they had undergone epithelial mesenchymal transition^10^. The mesothelial cell transformation and COX-2 level also correlated with MTAC^10^.

We found that peritoneal IL-6 and COX-2 levels are also closely linked. This finding is expected, because both cytokines are part of the NF-κB pathway downstream of common inflammatory triggers (e.g. IL-1β), which is involved in the peritoneal inflammation and fibrosis^18,19^. In addition, IL-6 production is directly induced by prostaglandin E2, the major metabolic product of COX-2, which may also serve as a positive feedback loop in the augmentation of NF-κB downstream signaling^20^.

In this study, PD effluent IL-6 and COX-2 levels in our overall cohort remained static (with a insignificant trend of decrease) after one year of PD. With careful subgroup analysis, PD effluent IL-6 and COX-2 levels significantly increased in patients with peritonitis, but decreased in those without peritonitis (see Fig. 1). The observation is distinctly different from previous report by the balANZ trial, which showed that dialysate IL-6 level increased from baseline to 24 month^8^. The reason of this discrepancy is unclear, but a large proportion of our cohort were low or low-average transporters with a low baseline D/P4, which may represent an intrinsic difference in the peritoneal membrane structure between our patients and the western population.

In this study, we did not find any correlation between PD effluent HGF level and peritoneal transport characteristics. Our result is different from the report of Bernardo *et al*.^21^, which showed that dialysate HGF levels significantly correlated with MTAC, especially amongst patients with ultrafiltration failure. However, the study of Bernardo *et al*. used the modified PET with 3.86% dextrose solution, recruited prevalent PD patients, and a substantial proportion of them had ultrafiltration problem^21^. The difference in enrollment criteria may explain the discrepancy in results. Our result, nevertheless, suggested that HGF may have a role in the modulation of peritoneal fibrosis following peritonitis. While it may not directly affect the peritoneal transport *per se*, previous studies suggested that HGF may prevent peritoneal fibrosis in response to external pro-inflammatory stimulus such as peritonitis^22^. HGF has been reported to be involved in preventing fibrosis in several organ tissues^23,24^, and HGF had been demonstrated to ameliorate peritoneal fibrosis in an animal model^11^.

A previous study by Witowski *et al*.^25^ showed that short term exposure to conventional PD solutions inhibits peritoneal mesothelial cell IL-6 and prostaglandin release. In our study, however, there was no significant change in PD effluent IL-6, COX-2, or HGF levels over one year, irrespective to the type of PD solution being used. It is possible that the sample size of our study is not adequate to detect a small change. Similar to the report by Cho *et al*.^8^, we also did not note any difference in PD effluent IL-6 or COX-2 levels between patients exposed to conventional and low GDP dialysis solutions.

In this study, baseline PD effluent cytokine levels did not predict the subsequent risk of peritonitis. It should be noted that the sample size of our study was small, and we only analyzed patients who remained on PD after one year. However, Cho *et al*.^8^ also reported previously that the risk of peritonitis was not predicted by baseline PD effluent IL-6 level. Similarly, the GLOBAL fluid study showed that systemic inflammation was associated with patient survival in PD patients, while intra-peritoneal inflammation was the determinant of peritoneal small solute transport but did not affect survival^17^. Their results suggested that intra-peritoneal and systemic inflammation are distinct entities. Unfortunately, we do not have any data on the serum level of corresponding cytokines.

Our study has several other limitations. First, the sample size was small and all patients came from a single center, so that our results may be prone to type 2 statistical error. Nonetheless, *post hoc* sample size estimation by the modified Piantadosi formula^26^ showed that to detect a correlation coefficient of 0.4 between PD effluent cytokine level and peritoneal transport characteristics with an alpha level of 0.05, a sample size of 47 would be sufficient.

Second, the duration of follow up was short, so that meaningful longitudinal change of peritoneal transport and the impact of peritonitis or prolonged exposure to unphysiological PD solutions may not be detected. Serial measurement of peritoneal cytokine and peritoneal functions for a longer duration would be needed in future studies.

In conclusion, we found that peritoneal IL-6 and COX-2 are important for the short-term control of peritoneal transport. Although PD effluent mediators assay may not be a suitable biomarker for clinical use, our result shed light on the physiological control and potential therapeutic targets for the modulation of peritoneal function.
